# Women's dietary diversity scores and childhood anthropometric measurements as indices of nutrition insecurity along the urban–rural continuum in Ouagadougou, Burkina Faso

**DOI:** 10.3402/fnr.v60.29425

**Published:** 2016-02-12

**Authors:** Takemore Chagomoka, Axel Drescher, Rüdiger Glaser, Bernd Marschner, Johannes Schlesinger, George Nyandoro

**Affiliations:** 1 Institute of Environmental Social Sciences and Geography, University of Freiburg, Freiburg, Germany; 2 Institute of Geography, Ruhr-University Bochum, Bochum, Germany; 3 Community Medicine Department, College of Health Sciences, University of Zimbabwe, Avondale, Harare, Zimbabwe

**Keywords:** women's dietary diversity score, anthropometric measurements, nutrition security, urban–rural continuum, Ouagadougou

## Abstract

**Background:**

Malnutrition is still prevalent worldwide, and its severity, which differs between regions and countries, has led to international organisations proposing its inclusion in the global development framework that will succeed the Millennium Development Goals (post-2015 framework). In Sub-Saharan Africa, malnutrition is particularly severe, among women and children under 5 years. The prevalence of malnutrition has been reported worldwide, differing from region to region and country to country. Nevertheless, little is known about how malnutrition differs between multiple locations along an urban–rural continuum.

**Objective:**

A survey was carried out in and around Ouagadougou, Burkina Faso, between August and September 2014 to map household nutrition insecurity along the urban–rural continuum, using a transect approach to guide the data collection.

**Design:**

Transects of 70 km long and 2 km wide directed radially from the city centre outwards were laid, and data were collected from randomly selected households along these transects. Women's dietary diversity scores (WDDSs) were calculated from a sample of 179 women of reproductive age (15–49 years) from randomly selected households. Additionally, anthropometric data (height/length and weight) of 133 children under 5 years of age were collected along the same transects for the computation of anthropometric indices.

**Results:**

We found that relative proportions of the nutrition indices such as stunting, wasting and underweight varied across the urban–rural continuum. Rural households (15%) had the highest relative proportion of WDDS compared with urban households (11%) and periurban households (8%). There was a significant association between children under 5 years’ nutritional status (wasting, stunting and underweight) and spatial location (*p*=0.023). The level of agricultural activities is a possible indicator of wasting in children aged 6–59 months (*p*=0.032).

**Conclusion:**

Childhood undernutrition certainly has a spatial dimension that is highly influenced by the degree of urbanity, which should be taken into consideration in policy formulation and implementation.

Malnutrition is severe, affecting lives of millions of people worldwide, mostly children and women (1). ‘Malnutrition is an abnormal physiological condition caused by inadequate, unbalanced or excessive consumption of macronutrients and/or micronutrients, it includes undernutrition and overnutrition as well as micronutrient deficiencies’ (2). Over 2 billion people are affected by micronutrient deficiency, also referred to as hidden hunger. On the other hand, more than 805 million people do not consume enough calories (3). According to the 2014 Global Nutrition report (1), 2–3 billion people are malnourished, diagnosed as undernourished, overweight or obese, or deficient in micronutrients. Furthermore, malnutrition is the major cause of mortality in children under 5 years old worldwide, with approximately one-third of the nearly 8 million deaths attributed to it (4).

Analysis of national data by Muthayya et al. (5) from 149 countries on stunting, anaemia and low serum retinol levels among infants and estimates of disability-adjusted life years (DALYs) indicated micronutrient deficiencies in 136 countries and revealed high levels of hidden hunger, stunting, iron deficiency anaemia and vitamin A deficiency in Sub-Saharan Africa.

Women's dietary diversity scores (WDDSs) have been proven to be a good measure of household macronutrient adequacy and household nutrition insecurity (6, 7). The WDDSs are based on a 24-h recall period and the number of food groups consumed and reflect the probability of micronutrient adequacy of the diet (7). In addition, anthropometric indices like weight for height (WHZ), especially of children under 5 years, are suitable to assess the nutritional status of children from birth to 59 months of age (8). Pinstrup-Andersen (9) pointed out that policies and programs aimed at improving child nutrition can be better informed by anthropometric measures than food security estimates. The 10-year United States Agency for International Development (USAID) multiple-sectoral nutrition strategy also identified both WDDS and anthropometric measurements as part of the 6 outcome level indicators (10). The Food and Agriculture Organization of the United Nations (FAO) et al. (11) proposed the use of the following indicators in the post-2015 framework: prevalence of stunting, prevalence of wasting, prevalence of overweight/obesity, prevalence of anaemia among women and children and the dietary diversity of women and infants. These indicators were proposed with the goal of ending all forms of malnutrition, with special attention to ending stunting (11). The FAO post-2015 framework was aimed at defining the global development framework, referred to as Sustainable Development Goals (SDGs) that succeeded the Millennium Development Goals (MDGs).

Although many studies have reported different measurements and prevalence of malnutrition in Burkina Faso (12–16), little is known about differences in malnutrition along the urban–rural continuum. Information on spatial variation on household nutrition insecurity is very useful in understanding the dynamics of household nutrition insecurity in various locations and helpful to resource allocation and targeting (17). Therefore, the objective of this study is to map household nutrition insecurity along the urban–rural continuum, in a transect approach, by analysing the variations in WDDS and anthropometric measurements among children under 5 years, as proven indices of nutrition security along the urban–rural continuum in Ouagadougou. This study also investigates the general association between households growing crops and keeping livestock, and the selected anthropometric measurements and WDDS. Studies from Burkina Faso confirm a moderately high prevalence of malnutrition in various age groups, mostly in children and women and mostly in urban and periurban areas. For example, a 40.4% prevalence of anaemia in children was reported in the urban and periurban areas of Ouagadougua (12, 15, 18). Several other studies have reported challenges related to the combination of fast-growing cities and food and nutrition insecurity (19–23). Many studies have also reported the involvement of urban households in agriculture in and around cities as a source of food and nutritious diets to cope with food and nutrition problems (24–27). Increased urbanisation has been associated with a decrease in biodiversity and loss of indigenous knowledge about the production and utilisation of indigenous species in urban areas (28–30).

## Materials and methods

### Description of study area

**Fig. 1:**
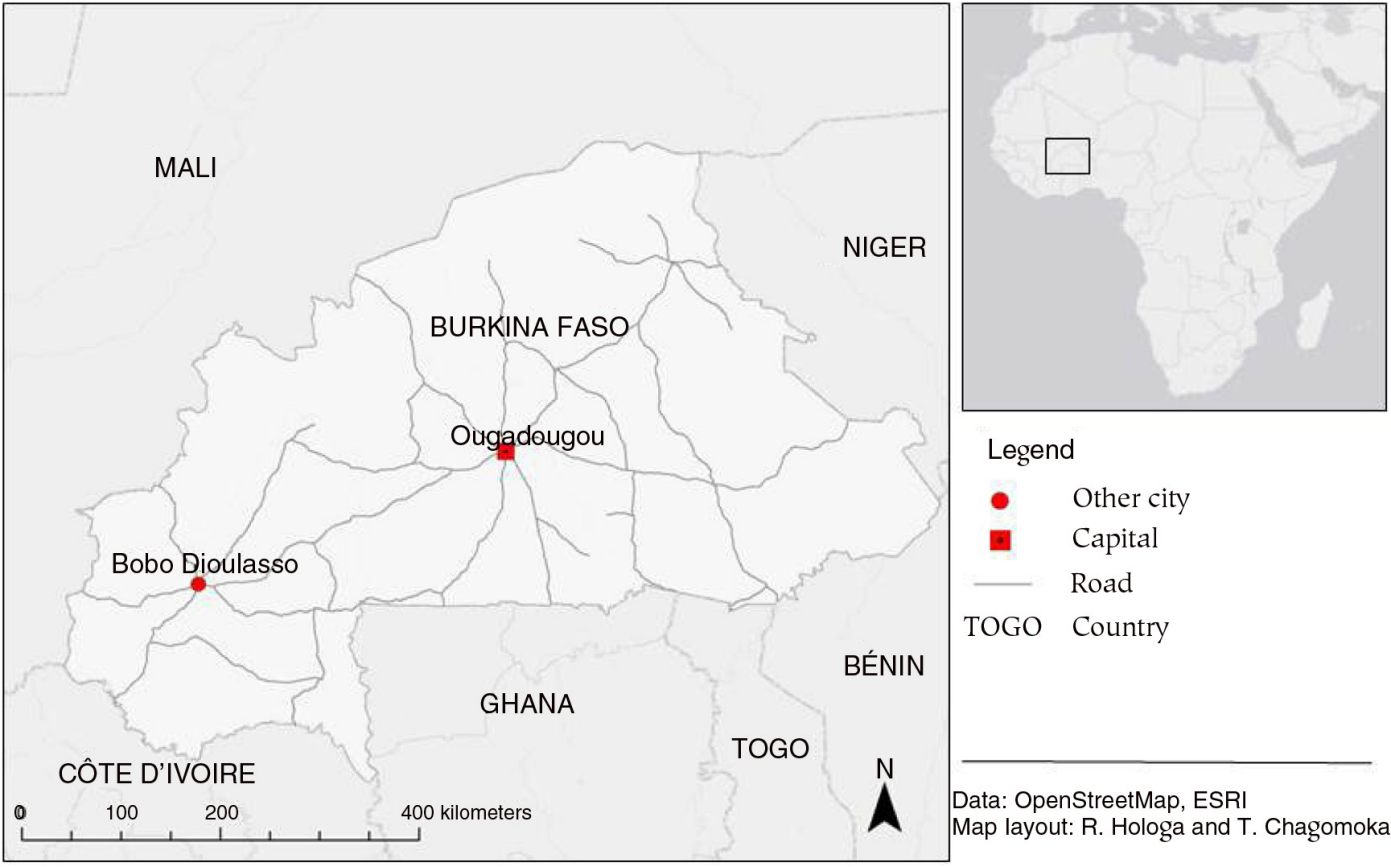
Study area location map.

Agriculture contributes significantly to the economy of Burkina Faso and employs more than 80% of the workforce (31). Burkina Faso is amongst the least developed countries in the world. In 2014, Burkina Faso was placed on number 181 out of 187 countries under the United Nations Development Programme (UNDP) Human Development Index (32). Ouagadougou (Fig. 1), the capital of Burkina Faso, is situated on the central plateau with an altitude of around 300 m above sea level. It is also the country's largest city, with a population of approximately 1,400,000 according to the 2006 census (33). Ouagadougou is situated in the Sudano-Sahelian climatic zone. Annual rainfall is around 800 mm, with the rainy season running from May to October. Heavy rainfall is usually experienced during the months of July and August (34).

### Study design and sampling approach

**Fig. 2:**
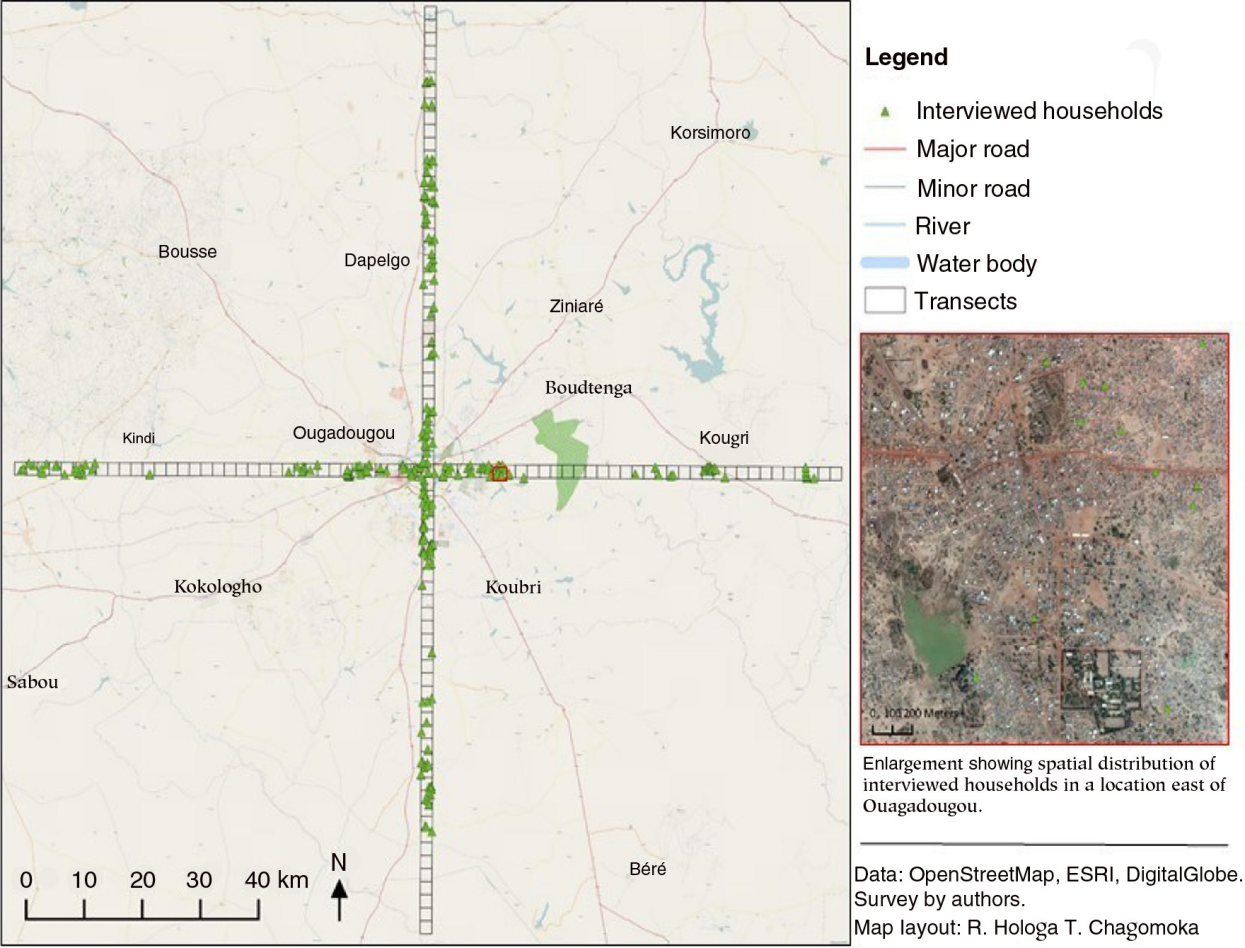
Transect approach.

This study is based on a cross-sectional and descriptive survey. A transect approach was used to guide data collection. Transects, 2 km wide and 70 km from Ouagadougou central market, were laid radially as shown in Fig. 2. Based on the relevant literature, working definitions of urban, periurban and rural areas were established (35–40). Within 10 km of the city centre was considered to be an urban area, 10–40 km as a periurban area and 40–70 km as a rural area. The work of Iaquinta and Drescher (41) strongly supported the identification of the periurban areas. Periurban areas have been reported to be complex zones, with rural people moving to urban areas looking for ‘places with better economic opportunities’ and, on the other hand, urban dwellers moving to periurban and rural areas to escape the scourge of urban poverty. All households along the transects were digitised and randomly selected, using Geographic Information Systems (GIS) (see Fig. 2). The study enrolled 20 households per zone, thus 3 zones by distance×4 sections of town×20 households=240. This translates to 80 households in each zone (urban, periurban and rural). The waypoints data were transferred to Global Positioning System (GPS), which helped in locating and identifying selected households. Selected householders who were not present during the visit and those who refused to take part in the survey or refused to give verbal consent were systematically replaced by taking the next household to the east, for consistency. This study sample size was calculated using the Dobson formula (42) to detect at least 3% of children aged 6–59 months old with severe acute malnutrition, as reported by Ministry of Health's Nutrition Department, National Nutrition Survey 2009 (43). Other parameters used were a 5% level of significance, a 5% error, a design effect of 1.5 and a 30% non-response rate to attain a minimum effective sample size of 88 children aged 6–59 months old. For triangulation, a minimum effective sample size of 88 women of reproductive age staying together with the enrolled children was set. This study uses data collected from 179 women of reproductive age (15–49 years) and 133 children under 5 years of age, staying in the sampled households to calculate WDDS and anthropometric indices. The survey targeted the youngest child of those under 5 years as an index child. The survey covered 32 districts^1^ in and around Ouagadougou.

### Data collection

This study uses data from a study conducted between August and September 2014 that had the objective of understanding the socio-spatial dynamics of household food and nutrition insecurity in Ouagadougou. A semi-structured questionnaire was used to collect information on both WDDS and anthropometric measurements. This study used WDDS elicited by FAO (7), based on a 24-h recall period. In total, 179 women of reproductive age between 15 and 49 years were interviewed to obtain data on the WDDS. Additionally, anthropometric measurements of the heights and lengths of 133 children under 5 years of age, staying together with women of reproductive age in the sampled households, were collected. A SECA floor electronic scale, model 881 U, was used to record the weight of the children, while the heights and lengths of the children were measured by a stadiometer (a light portable wooden board with a graduated tape measure). The recumbent lengths of children between 6 and 23 months were taken while the children were lying on their backs, whereas the heights of children between 24 and 59 months were taken while standing (44). Weights of children between 6 and 59 months were taken. For children who could not stand on the scale on their own, the mother/baby weight recording approach was used as discussed by Cogill (44). The digital scale was standardized by measuring various objects with standard weight, for example, 5-kg bags of rice from various sources, just to ensure that the scale was taking correct measurements. The assistant was trained to take anthropometric measurements before pre-testing, based on the work of Cogill (44). Pre-testing of the tools was done before the data collection for this study commenced. In order to determine the relation between the nutritional status and agricultural activities of the households, information on the households’ crop production and use (crops gown, quantities sold and quantities consumed) and livestock keeping and use (animals kept, quantities sold and quantities slaughtered for household consumption) was collected. All data were collected between August and September 2014 by the first author and trained assistant to ensure quality data and consistency. Data collection took place at the location of randomly selected households, as directed by GPS.

### Nutrition indicators used in the study

The study used 4 indicators of nutrition. First, the WDDS was used as a proxy of household dietary diversity, such that the higher the WDDS, the higher the dietary diversity (ranging from 0 to 9) (7). Based on food items consumed in the past 24 h, respondents were assigned the number of food groups they consumed, ranging from 0 to 9. Households were classified into 3 groups, based on the distribution in the sample as recommended by FAO (7): ≤3 food groups as lowest dietary diversity, 4–5 food groups as medium dietary diversity and ≥6 as high dietary diversity.

The second indicator, stunting (<−2 *z*-score), is inadequate length or height relative to age (and reflects chronic malnutrition) and is usually due to long-term nutritional deprivation (45, 46). Third, wasting (<−2 *z*-score) is inadequate weight relative to length or height (and reflects acute malnutrition) and is usually due to insufficient food intake or a high incidence of infectious diseases like diarrhoea (45, 46). Finally, underweight (<−2 *z*-score) is inadequate weight relative to age (and reflects both chronic malnutrition and acute malnutrition) (45, 46).

### Data management and analysis

Data were entered in Epidata 9 software and then exported to Stata 12 for cleaning and analysis. Categories for agricultural production were generated (livestock only, crops only, crops and livestock, and nothing) and tested for association against nutrition indicators for both women and children, separately. Chi-square tests were run to determine the presence and significance of the association after categorising nutrition status indicators. We tested for the association between either crop production or livestock production versus nutrition status. The anthropometric data of children aged 6 and 59 months were entered and analysed in WHO Anthro v.3.2.2 (47).

### Ethical considerations

The study objectives and purpose were made clear to community leaders, household heads and respondents. Permission was sought before data collection from local leaders, and respondents gave verbal consent, which was recorded as part of the questionnaire responses. The data from all respondents were treated with confidentiality. Respondents had the opportunity to stop participating in the research at any time of their choice during interviews. None of the participants who gave consent at the beginning of the survey opted to stop.

## Results

The results represent the findings of a household survey carried out between August and September 2014 in and around Ouagadougou.

### Demographic characteristics overview of the study

This study is part of a research project carried in Ouagadougou with the objective of understanding the dynamics of urban, periurban and rural agriculture and its association with household food and nutrition insecurity. Table 1 shows the general picture of demographic characteristics of the groups enrolled in the study.

**Table 1:** Demographic characteristics of respondents

Characteristics		Urban%(*n*=80)	Periurban%(*n*=80)	Rural%(*n*=80)	Total% (*n*=240)
Gender	Men	37	63	56	52
	Women	63	37	44	48
House with agriculture	Yes	13	76	99	63
	No	87	24	1	37
Age class of respondents	≤20 years	4	0	1	2
	21–59 years	87	86	79	84
	≥60 years	9	14	20	14
Level of education	None	38	55	71	55
	Primary	22	28	15	22
	Secondary	29	6	6	14
	Tertiary	10	4	3	5
	Koranic	1	7	5	4
Household religion	Muslim (m)	58	50	30	46
	Christian (c)	39	46	50	45
	Tradition (t)	1	3	13	5
	Mix m+c	1	0	4	2
	Mix m+t	0	0	1	0.4
	Mix c+t	0	1	1	0.8
	Mix all	1	0	1	0.8
Ethnic group	Mossi	75	93	96	88
	Samo	6	3	0	3
	Gourounsi	4	1	1	2
	Bissa	4	2	0	2
	Fulani	1	1	3	2
	Others	10	0	0	3

Source: Ouagadougou August–September 2014 survey (48).

### Consumption distribution based on 9 food groups

Out of the 9 identified food groups (see Table 2), 98% of respondents were found to consume food items from the starchy samples group along the urban–rural continuum. More households in rural areas (80%) consumed more of the green leafy vegetables, compared with households in periurban areas (56%) and urban areas (45%). Of this group, most consumed dark leaf vegetables that included baobab leaves (*Adansonia digitata*), cowpea leaves (*Vigna unguiculata*), jute mallow (*Corchurus olitorious*), bean leaves (*Phaseolus vulgaris*), onion leaves (*Allium cepa*) and roselle leaves (*Hibiscus sabdariffa*). Rural households also consumed more (78%) of the food items from the group, other vitamin A fruits/vegetables, compared with periurban households (59%) and urban households (56%). Sumbala (*Parkia biglobosa*) and shea nut tree fruits (*Vitellaria paradoxa*) were the most consumed food items under the other vitamin A fruits and vegetables group. On the other hand, urban household consumed more (85%) of food groups from other fruits/vegetables compared with periurban households (69%), *p*=0.033 and rural households (67%), *p*=0.020. Urban households also consumed more legumes (52%) compared with periurban households (32%), *p*=0.024, and rural households (30%), *p=*0.015 (Table 2).

**Table 2:** Consumption distribution based on 9 food groups

Location		Urban% (*n*=66)	Periurban% (*n*=59)	Rural% (*n*=54)	Totals% (*n*=179)
Food groups	Starchy samples	98	98	98	98
	Dark green leafy vegetables	45	56	80	59
	Other Vitamin A fruits/vegetables	56	59	78	64
	Other fruits/vegetables^a^	85	69	67	74
	Organ meat	0	0	2	1
	Flesh meat	73	73	63	70
	Eggs	2	3	4	3
	Legumes^b^	52	32	30	39
	Milk	3	2	0	2
WDDS ≤3	30	32	25	30	

a Urban versus periurban (*p*=0.033); urban versus rural (*p*=0.020); periurban versus rural (*p*=0.380).

b Urban versus periurban (*p*=0.024); urban versus rural (*p*=0.015); periurban versus rural (*p*=0.819).

### Distribution of WDDS along the urban–rural continuum

**Fig. 3:**
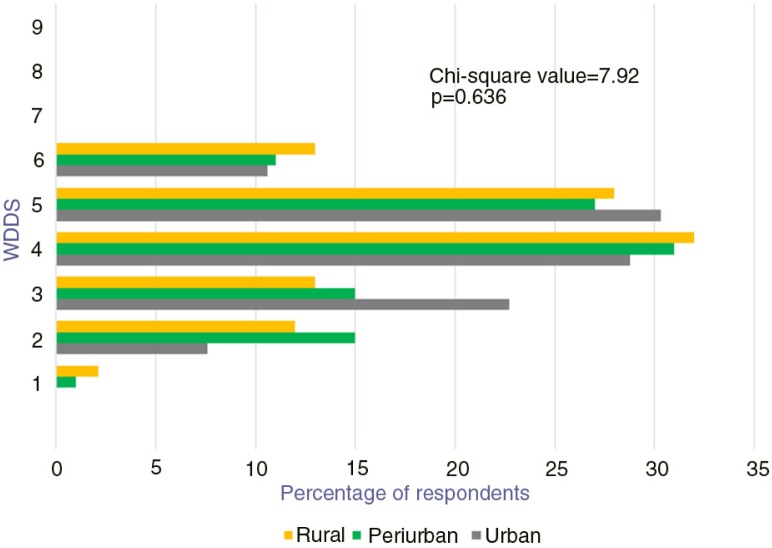
Distribution of WDDS along the urban–rural continuum.

Along the urban–rural continuum, none of the households consumed as much as 7–9 food groups (Fig. 3), revealing a general limited diversity of diets. The WDDS was low mostly in urban areas compared with periurban and rural areas. Less than 10% of the households consumed more than 5 food groups and only 20% of the households consumed up to 5 food groups. On the other hand, only few households in urban and periurban areas consumed only one food group during the reported period.

### WDDS and anthropometric measurements as indices of household nutrition along the urban–rural continuum

There were variations in the prevalence of different nutrition indices along the urban-rural continuum (Table 3). Nevertheless, only wasting was statistically significant (*p*=0.046) along this continuum, with more children under 5 years in urban areas (16%) wasted compared with periurban areas (6%) and rural areas (2%). Rural households (15%) had the highest relative proportion of WDDS compared with urban households (11%) and periurban households (8%). There was high relative proportion of stunting in periurban areas (31%) compared with rural areas (29%) and periurban areas (11%). Periurban areas also had the highest relative proportion of underweight (19%) compared with rural areas (15%) and urban areas (5%). Nevertheless, the differences in WDDS, stunting and underweight along the continuum were not statistically significant (Table 3).

**Table 3:** Women and children nutrition status across the rural–urban continuum

	Indicators	Urban%	Periurban%	Rural%	*p*
Women	Sample size (*n*)	(*n*=66)	(*n*=59)	(*n*=54)	
	WDDS ≥6 (highest food diversity)	11	8	15	0.555*
	WDDS<6 (medium and lowest food diversity)	89	92	85	
Children	Sample size (*n*)	(*n=*37)	(*n*=48)	(*n*=48)	
	Wasted (WHZ<−2 SD)^a^	16	6	2	0.032**
	Stunted (HAZ<−2 SD)^a^	11	31	29	
	Underweight (WAZ<−2 SD)^a^	5	19	15	

* Tested by Pearson chi-square (expected cell count>5);

** tested by Fischer exact (expected cell count<5).

a There was a statistically significant association at 95% confidence level between child wasting, stunting and underweight and their location (rural, periurban and urban).

### Association between livestock production, crop production and nutrition indicators

Engagement in agricultural activities, that is, crop production and livestock keeping, varied along the urban–rural continuum, with 13% of household in urban areas, 76% in periurban areas and 99% in rural areas. Urban households had the highest relative proportion of wasting (16%) compared with periurban households (6%) and rural households (2%) (Table 3). The level of agricultural activities is a possible indicator of wasting in children aged 6–59 months (*p*=0.032) (Table 4).

**Table 4:** Association between livestock, crop production and nutrition indicators

	Nutrition status indicators							
	
	Women		Children					
		
	WDDS ≥6 Dietary diversity		HAZ<−2SD Stunting		WHZ<−2SD Wasting		WAZ<−2SD Underweight	
				
Agriculture production	Yes	No	Yes	No	Yes	No	Yes	No
Livestock only	3	20	4	13	2	15	3	14
Crops only	0	6	2	3	0	5	1	4
Crops and livestock	12	89	25	62	3	84	13	74
Nothing	5	43	2	21	5	18	1	22
*p*	0.823		0.210		0.023*		0.529	

* Statistically significant at 95% level of confidence.

## Discussion

Dietary diversity is one of the strategies proposed to tackle the scourge of micronutrient malnutrition by many organizations including World Health Organization (WHO), the FAO of the United Nations and HarvestPlus (49, 50). Other strategies to prevent micronutrient malnutrition include food fortification, supplementation and the biofortification of staple crops or an agriculture-based strategy (49, 50). The pros and cons of the different strategies have been discussed. For example, the use of encapsulation options in iron fortification was reported to increase the price of the fortified product by as much as 30% (49).

However, our study results show that most households in and around Ouagadougou relied mostly on starchy-based diets, with 98% of households consuming this food group. This is different from what was observed in Ethiopia, where rural households in Ethiopia were consuming more starchy-based food than urban households (51). Nevertheless, this result confirms, more or less, what was observed before in Ouagadougou (14). The high consumption of starchy diets could be explained by the high production of starchy-based cereal crops like pearl millet (*Pennisetum glaucum*), sorghum (*Sorghum bicolor*) and maize (*Zea mays*) that are often used to prepare staple traditional dishes like *Tô* (cooked millet, sorghum, or corn meal), *Zoomkoom* (a soft drink made from millet flour) and *Degue* (a drink made from pearl millet and yogurt).

Households in rural areas were consuming more dark green leafy vegetables compared with households in periurban areas and households in urban areas. These dark green leafy vegetables include baobab leaves, cowpea leaves, jute mallow, bean leaves, onion leaves and roselle leaves. Rural households consume more dark green leafy vegetable than urban households and periurban households because these vegetables are available naturally (e.g. baobab) and through subsistence cultivation (e.g. cowpea leaves and roselle leaves). Work of Mertz et al. (52) noted the high usage of baobab leaves, onion leaves and jute mallow in Burkina Faso. Dark green vegetables are recommended as a good source of vitamin A (7). Vitamin A deficiency is one of the most challenging form of micronutrient deficiency, with serious effect like night blindness and cognitive impairment, mostly among children under 5 years and pregnant women (1, 45). Jute mallow, one of the most commonly consumed leafy vegetables, was reported to have high levels of iron (53, 54). Iron deficiency is the cause of about 50% of all cases of anaemia (55). This makes jute mallow a potential source of iron for vulnerable groups, such as children under 5 years and pregnant women.

The results show a general limited dietary diversity, with less than 15% of the households consuming more than 5 out of the 9 food groups. None of the households consumed between 7 and 9 food groups. Becquey et al. (14) reported that the diets in Ouagadougou were mainly made of cereals, vegetables and fats from vegetable sources. The FAO (7) highlighted that WDDS food groups better reflect micronutrient intake than economic access to food. The regular consumption of dishes covering only a limited food groups may translate to stunting, wasting and underweight. Rural households had the highest dietary diversity compared with urban and periurban households, due to subsistence farming and the availability of natural resources like baobab trees. Interestingly, rural households that had the highest proportion of WDDS had also the lowest prevalence of wasting (WHZ<−2 SD).

There was limited consumption of organ meat in the study sample, with only 2% of the households in rural areas reported to have consumed this food group and none in both urban areas and periurban areas. Organ meat falls in the food groups that are sources of haem iron, which is more bioavailable than non-haem iron and also enhances the absorption of the non-haem iron present in the same meal (7). Organ meats (liver, kidney, heart, or blood-based foods) are the richest source of haem iron. Iron deficiency anaemia is considered a public health problem and can affect the immune status and morbidity from infections of all age groups, and increases perinatal risks for mothers and neonates (56). The work of Daboné et al. (12) in Ouagadougou, based on haemoglobin (Hb) concentration to assess anaemia in school children in urban and periurban areas, reported a 40.4% prevalence of anaemia.

Study results show variability in different nutrition indices along the urban-rural continuum. There was a significant association between children under 5 years’ nutritional status (wasting, stunting and underweight) and spatial location. These results support the growing evidence of urban poverty and its associated challenges of food and nutrition insecurity in most cities in Sub-Saharan Africa (57–59). Wasting reflects acute malnutrition and is usually due to insufficient food intake or a high incidence of infectious diseases like diarrhoea (45). There was also high relative proportion of stunted children in periurban areas, compared to urban and rural areas. Daboné et al. (12) also reported higher levels of thinness and stunting in periurban compared to urban schools in Ouagadougou.

The level of agricultural activities is a possible indicator of wasting in children aged 6–59 months. Improving agricultural activities is likely to help in addressing wasting of children aged 6–59 months. On the other hand, urban households had the highest significant prevalence of wasting, compared to periurban areas and rural areas. Zezza and Tasciotti (60) reported consistent evidence of a positive statistical association between engagement in urban agriculture and dietary adequacy indicators. Drescher and Iaquinta (61) highlighted that the challenges of rapid growing cities and food shortages often lead urban dwellers into farming so to feed their families. The results of this study suggest that the engagement of urban households in crop production and livestock keeping significantly helps to reduce wasting.

This study is based on transect approach. The advantage of this approach is the probability of including households that may be excluded in most sampling approaches, which usually follow the linear settlement pattern of households along developments like major roads. The weakness of this approach is the concentration along the transects and not elsewhere.

## Conclusions

The study results reveal that malnutrition certainly has a spatial dimension that is highly influenced by the degree of urbanity. Furthermore, it could be shown that WDDS and anthropometric measurements are useful indices of household nutrition along the urban–rural continuum. There was high consumption of starchy staple crops like sorghum, pearl millets, cassava, yams and maize. At the same time, there were soaring levels of wasting and stunting among children under 5 years of age, mostly in periurban areas. Therefore, the introduction of vegetables, especially traditional vegetables, which are known to be rich in micronutrients including vitamin A and iron (62, 53), will help to diversify diets along the urban–rural continuum. Further research is needed to identify the appropriate mechanisms to achieve this diversification. There was a moderately significant prevalence of wasting in urban areas, compared with periurban areas and rural areas. This result confirms the growing evidence of urban poverty related to challenges like food and nutrition insecurity in and around cities in Sub-Saharan Africa. There was also high relative proportion of stunting in periurban areas, compared with urban areas and rural areas. This reveals the complexity of periurban areas that is often associated with an influx of people from rural areas and urban areas, which are perceived as a safety net.

The study shows a general low diversity of household diets along the urban–rural continuum, with fewer than 10% of households consuming 5 food groups. Nevertheless, the availability of some traditional vegetables like jute mallow and trees like baobab played a critical role in the study area as sources of micronutrients, as they formed an important part of the surveyed households’ diets. Rural households had the highest dietary diversity compared with urban and periurban households.

Most households relied on starchy-based food staff across the urban–rural continuum. Sorghum, pearl millet and maize formed important parts of the starchy-based cereals often used in the study area and consumed as traditional processed dishes.

Households who were producing crops and keeping livestock in the study area were associated with the reduction of wasting amongst children under 5 years of age compared with those not producing crops or not keeping livestock. Urban households had the highest significant prevalence of wasting. Therefore, the involvement of urban households in crop production and livestock keeping will reduce wasting significantly.

## Footnotes

1 Districts 1, 2,3,4,5, 8, 9, 10, 11, 12, Baskuy, Dapelgo, Dawelge, Doulougou, Ipelce, Kindi, Kombissiri, Komsilga, Mogtedo, Nagreongo, Nandiala, Nongremassom, Ourgou-Manega, Paabre, Roulougou, Saaba, Sapone, Sourgoubila, Tanghindassouri, Zam, Ziniare, Zorgho.
